# Fractal-Like Flow-Fields with Minimum Entropy Production for Polymer Electrolyte Membrane Fuel Cells

**DOI:** 10.3390/e22020176

**Published:** 2020-02-04

**Authors:** Natalya Kizilova, Marco Sauermoser, Signe Kjelstrup, Bruno G. Pollet

**Affiliations:** 1 Warsaw University of Technology, Institute of Aviation and Applied Mechanics, 00-665 Warsaw, Poland; 2 Department of Applied Mathematics, V.N. Karazin Kharkov National University, 61022 Kharkiv, Ukraine; 3 PoreLab, Department of Chemistry, Norwegian University of Science and Technology, 7491 Trondheim, Norway; marco.sauermoser@ntnu.no (M.S.); signe.kjelstrup@ntnu.no (S.K.); 4 Department of Energy and Process Engineering, Norwegian University of Science and Technology, 7491 Trondheim, Norway; bruno.g.pollet@ntnu.no

**Keywords:** minimum entropy production, fuel cell, flow field plate, fractal channels

## Abstract

The fractal-type flow-fields for fuel cell (FC) applications are promising, due to their ability to deliver uniformly, with a Peclet number Pe~1, the reactant gases to the catalytic layer. We review fractal designs that have been developed and studied in experimental prototypes and with CFD computations on 1D and 3D flow models for planar, circular, cylindrical and conical FCs. It is shown, that the FC efficiency could be increased by design optimization of the fractal system. The total entropy production (TEP) due to viscous flow was the objective function, and a constant total volume (TV) of the channels was used as constraint in the design optimization. Analytical solutions were used for the TEP, for rectangular channels and a simplified 1D circular tube. Case studies were done varying the equivalent hydraulic diameter (D_h_), cross-sectional area (D_Σ_) and hydraulic resistance (D_Z_). The analytical expressions allowed us to obtain exact solutions to the optimization problem (TEP→min, TV=const). It was shown that the optimal design corresponds to a non-uniform width and length scaling of consecutive channels that classifies the flow field as a quasi-fractal. The depths of the channels were set equal for manufacturing reasons. Recursive formulae for optimal non-uniform width scaling were obtained for 1D circular D_h_ -, D_Σ_ -, and D_Z_ -based tubes (Cases 1-3). Appropriate scaling of the fractal system providing uniform entropy production along all the channels have also been computed for D_h_ -, D_Σ_ -, and D_Z_ -based 1D models (Cases 4-6). As a reference case, Murray’s law was used for circular (Case 7) and rectangular (Case 8) channels. It was shown, that Dh-based models always resulted in smaller cross-sectional areas and, thus, overestimated the hydraulic resistance and TEP. The D_Σ_ -based models gave smaller resistances compared to the original rectangular channels and, therefore, underestimated the TEP. The D_Z_ -based models fitted best to the 3D CFD data. All optimal geometries exhibited larger TEP, but smaller TV than those from Murray’s scaling (reference Cases 7,8). Higher TV with Murray’s scaling leads to lower contact area between the flow-field plate with other FC layers and, therefore, to larger electric resistivity or ohmic losses. We conclude that the most appropriate design can be found from multi-criteria optimization, resulting in a Pareto-frontier on the dependencies of TEP vs TV computed for all studied geometries. The proposed approach helps us to determine a restricted number of geometries for more detailed 3D computations and further experimental validations on prototypes.

## 1. Introduction

Fossil fuels compose the main part of the world energy production (>80% in 2016) [1]. The negative impact caused by this on the environment and biosphere of the planet has now been documented, and a transition to green technologies is in demand. It is, however, known that efforts to increase the energy conversion efficiency are equally important for a reduction of CO_2_ pollution levels in the atmosphere, as replacements technologies like solar, thermal, geothermal, tidal, and photovoltaics are not sufficient. Methanol, hydrogen, and other types of fuel cells (FCs) are direct convertors of chemical to electric energy, and play a central role in the vision of the hydrogen society [2]. The polymer electrolyte membrane fuel cells (PEMFC) are also attractive because of their portability and scalability, low operating temperature and high current density (i.e., high performance). A major aim for PEMFC development is thus to increase the efficiency and reduce the production costs. The latest report of the US Department of Energy (25 April 2018), target a price of $40/kW at 500,000 systems per year, including 80 kW automobiles and 160 kW trucks [3].

**Figure 1:**
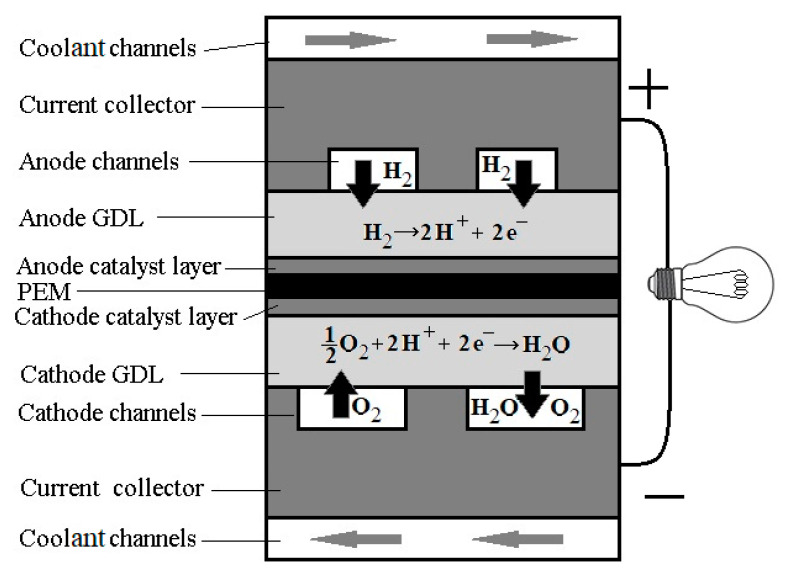
Components of PEM FC. A schematic representation showing different layers. The channels for gas transport are engraved in the flow field, see white cross-sectional area of the channels.

One of the important components of the FC are the anode and cathode flow-field plates (FFP). These deliver humidified reactant gases (H_2_ and O_2_) to the cell and remove excess water from the catalytic layer (Figure 1). Quite a big number of different designs of FFPs have been proposed and tested, using computational fluid dynamics (CFD) and experimental methods (see review [4]). The FFP comprises >60% of the weight and 30% of the total cost of the FC stack [5,6] and its optimization could improve the FC performance significantly [7]. 

Flow field optimization problems are well known from fluid-based micro-coolers or heaters. Permanent miniaturization of electronic devices can lead to high local rates of energy dissipation [8]. The temperature inside an electronic device can rise far beyond that allowed for proper unit performance. Since Tuckerman proposed his system of micro-channels for cooling of integrated circuits in 1981 [9], many similar designs have been proposed and proven to give superior performance, also in FCs, see [4]. Sauermoser et al. [4] concluded their review, pointing out that among flow-fields tested so far, the fractal-like fields seemed to hold most promise.

The aim of this paper is to examine fractal-like flow field designs and find an optimal one satisfying the minimum entropy production in the FFP. Entropy is a quantitative measure of any physical, chemical or biological process, and the minimum entropy production approach was shown to be an efficient optimization criterion for different natural systems and engineered units [10]. As it will be shown in the next paragraph, 3D CFD computations of the flow field uniformity are usually used for FFP optimization via dimensions and cross-sectional area of the channels. However, 3D simulations could be used to compare efficiency of several designs only. In this paper we carry out numerical computations on simplified 1D models that allows direct semi-analytical mathematical solution of the optimization problem including optimization criteria and constraints. This simplifies the design process in terms of computer costs, efforts and speed. We first examine the nature of tree-like branching networks and review their flow properties. We establish a reference system, defined by the equations for fluid flow in networks. The well-known Murray’s law will be used for this purpose. We next formulate the optimization problem, with objective function and corresponding constraint. For the simplest flow pattern, there is a 1D-analytical solution to the optimization problem (Section 2). Approximations to this solution can be found in terms of a solution for a constant pressure gradient. The 1D-solution was validated using more detailed 3D-computations. This is also described in Section 4. This means that a 1D model of the fluid transport in the fractal-like flow field can be found and trusted for simplified studies of the performance of fractal-like distributor flow fields under various conditions.

## 2. Tree-Like Branching Networks for Distribution of Fluids to the Catalytic

### 2.1. Properties of Natural and Man-Made Distribution Systems

Complex networks of rigid and elastic tubes are abundant in Nature. We find them in blood and in lymphatic vessels, conducting pathways for plant saps in plant roots, stems and leaves, fluid trophic systems in animals, respiratory systems, and many others [10,11,12,13,14]. Such branching networks are often fractal-like. Different scaling factors have been observed, many corresponding to optimal network performance (minimum energy dissipation for transport and maintenance) [10,15]. Anticipating that such fields can be useful for FCs, we describe first the characteristics of such natural and man-made systems. The overview will later help us choose variables for FFP optimizations.

Regularities in the structure of arterial vasculatures have been in situ measured and formulated as a relationship between the vessel diameter and branching angles W. Roux [16]. The corresponding mathematical relations were obtained almost 50 years later by C.D. Murray. He found that the vessel diameters in the bifurcation obeyed the relationship [17]

(1) $$d_{0}^{\gamma }=d_{1}^{\gamma }+d_{2}^{\gamma },$$

and the branching angles $\alpha_{1},\alpha_{2}$ obeyed the relationship [18]

(2) $$\varphi_{1}^{}=\arccos\left(\frac{{\left(1+\xi_{}^{\gamma }\right)}^{4/\gamma }+1-\xi_{}^{4}}{2{\left(1+\xi_{}^{\gamma }\right)}^{2/\gamma }}\right),\;\varphi_{2}=\arccos\left(\frac{{\left(1+\xi_{}^{\gamma }\right)}^{4/\gamma }+\xi_{}^{4}-1}{2\xi_{}^{2}{\left(1+\xi_{}^{\gamma }\right)}^{2/\gamma }}\right),$$

where $d_{0}^{},d_{1}^{},d_{2}^{}$ are the diameters of the parent and the two daughters’ vessels in the bifurcation, γ = const, and $\xi =\min\{d_{1},d_{2}\}/\max\{d_{1},d_{2}\}$ is the branching asymmetry.

A property is fractal when the diameters and lengths of the tubes are related to the dimensions of the feeding tube (0-th generation order) by the scaling laws, here

(3) $$d_{j}=d_{0}a^{-j},\;L_{j}=L_{0}b^{-j}$$

where *a* and *b* are constants (scaling factors) for diameters and lengths. The relationships (1) and (2) are solutions to an optimization problem that is seeking minimum entropy production due to energy dissipation in a system of fixed total volume (TV) [17,18]

(4) $${\dot{S}}_{irr}\to \min,\;V=const.$$

Equations (1) and (2) were documented for arterial [11,19,20,21,22,23], and venous [11,19], respiratory [24,25] systems in mammals, astrorhizal systems in sponges [11], tree trunks and shoots [14,26,27], plant leaves of different types [15,27,28]. The power in (1) was estimated as *γ* = 2.55 − 3.02 for arterial, γ=2.76−3.02 for venous, γ=2.61−2.91 for respiratory systems, γ≈3 for plant leaves [15,28] and tree branches [27]. The average value γ= 3 was found for relatively slow laminar flows, while γ~2.33 was obtained for turbulent flows in larger arteries and airways with high Reynolds numbers Re > 1000 [29]. In small vessels, where the non-Newtonian properties of blood must be taken into account, γ~2.92 [15]. 

Note, the optimization problem formulation (4) can be understood in two ways: the objective is minimum entropy production for a given volume, or alternatively, minimum TV for a given entropy production

(5) $$V\to \min,\;{\dot{S}}_{irr}=const.$$

Both objective functions, with their corresponding constraints, provide the same mathematical or numerical solution.

The Murray’s optimal design (1), (2) has been used in many technical and biomedical units [10], such as drainage systems, porous filters [30,31], flow fields for heating of liquids in solar panels, microelectronic coolers, fluid/gas distributors, microfluidic manifolds for lab-on-a-chip units [32], and FFPs and cooling systems for FCs [4,33,34]. In this study we use Murray’s law (1) with γ=3 as a reference case. 

The present study is building on a recent review [4] on conventional, synthetic, biomimetic, and other designs of fuel cell flow fields. Table 1 adds a comprehensive overview of papers on fractal-like micro-channels with different bifurcation angles $\alpha \equiv \alpha_{1}+\alpha_{2}\in [75;180]$°. The angles $\alpha_{1,2}$ = 37.5° correspond to symmetrical branching according to (2). The designs have been studied experimentally for Reynold’s numbers, Re = 150–1200 [35]. The dimensions of the first channel were *L* = 4 mm, *h* = 0.125 mm, *w* = 0.125, 0.250, 0.375 mm (*L* × *h* × *w* = length × depth × width; aspect ratios *AR* = h/w = 1, 1/2, 1/3). The diameter of the tubes in the generations j=0,1,2,… was computed from Murray’s law (1), giving diameter ratio $d_{j+1}/d_{j}=2^{-1/3}$, and length ratio $L_{j+1}/L_{j}=2^{-1/2}$. This ratio was used in a majority of the studies listed in Table 1, possibly because the length scaling $L_{j+1}/L_{j}=2^{-1/3}$ produces overlapping networks starting from some generation order [36,37,38].

Note that two different design concepts for the fractal FFPs have been studied:

1) Open-side channels which are in direct contact with a gas distribution layer (GDL);
2) Closed channels which are in direct contact with GDL via the branches of the last generation only.

Open-side channels provide non-uniform distribution of the reactant gases and temperature, and their optimization can be carried out in direct connection to thermal, chemical and electric phenomena in the membrane electrode assembly (MEA) of FC. The closed fractal flow-fields provide the most uniform flow and temperature distribution (Table 1). When the shape, size, location and the flow velocity at the outlets are given, the FFP can be optimized independently on the MEA.

Experimental results have shown that fractal tree-like micro-channels have a much higher heat transfer coefficient than straight micro-channels for the same heat transfer area, but they also have a higher pressure loss because of vortexes generated at the channel branching points. The aspect ratio was found to be essential for system performance; the highest performance coefficient $PC=J_{H}/(\Delta P\cdot \dot{V})$ where $J_{H}$, ΔP, and $\dot{V}$ are the heat flux, pressure drop and flow rate, was obtained at *AR* = 1/3. The pressure loss increased with the Re number. For such flow regimes, semi-circular shapes at the bends and bifurcations were recommended [35].

For rectangular channels, Murray’s law has been applied, using hydraulic diameter ratios $D_{h,j+1}/D_{h,j}=2^{-1/3}$. The corresponding cross-section ratio of the adjacent channels was related as $A_{j+1}/A_{j}=2^{-2/3}$. In real rectangular channels [37,38] the ratio

(6) $$w_{j+1}/w_{j}=2^{-1/3}$$

was used with cross-section ratio $A_{j+1}/A_{j}=2^{-1/3}$. This introduces differences in measured and computed data for 3D CFD and simplified 1D models.

Different fractal tree-based designs for square, circular and cylindrical FFPs based on Murray’s law (1), (2), computed and tested so far, are summarized in Table 1. The table shows that fractal-like designs lead to a decrease in the pressure drop that drives the fluid through the distributor, and therefore in the total dissipation, and this fact has motivated the present work. The lengths of the channels of the uniform flow distribution system must be determined by the size of the membrane. They cannot satisfy the fractal scaling relation (2), however. This makes the optimization different from conditions that give Murray’s law (1). We shall see that the optimization formulations (4) and (5) with added constraints can be used to define trade-off conditions (Pareto-frontiers) for possible geometric variables in the FFP design. 

**Figure 2:**
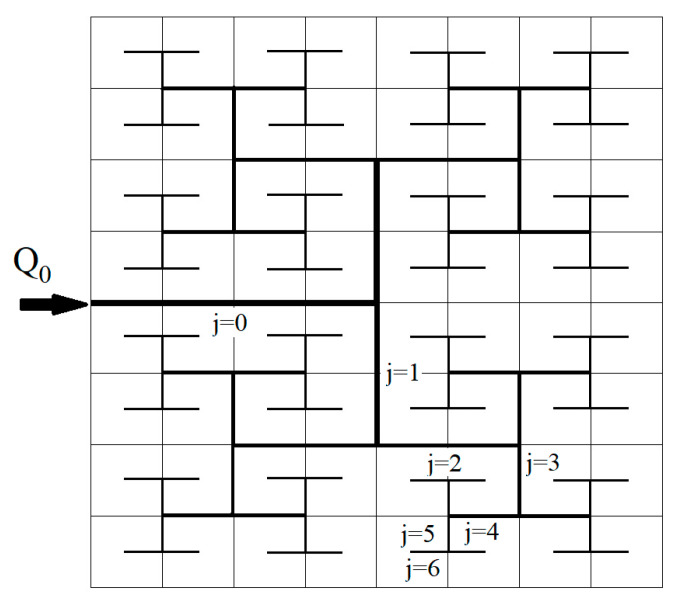
A symmetrical fractal-like flow field for uniform delivery of reactants to a catalytic layer. The numbering of generations of branches is shown.

The very first fractal FFP for FCs was proposed in [42] (Figure 2) based on a few combinations of fractal and parallel designs. The ratios of the diameters in the junctions were not optimized in this design. On a prototype with 16 parallel rectangle channels, it was shown, at low inlet flow velocity *v* = 1.4 m/s, that the flow difference in the terminal channels with smallest size was <4%, while at the velocities *v* = 7–20 m/s it was ≥12% [65] that implies high flow non-uniformity along the FFP. Later different types of fractals in combinations with conventional serpentine, parallel, pin-type, and interdigitated designs were developed and tested (see review [4]). During the last decade, the fractal distributors with branching angle $\alpha =180^{\circ }$ with sharp and smoothed T-junctions have become the most attractive designs for the FFPs for FC [66,67,68]. The distributors with smoothed T-junctions need ~15.9%–25.1% lower pressure drop than the similar ones with sharp junctions [51]. Numerical simulations revealed significant pressure losses in single L-bends and T-junctions [69], while in the smoothed junctions improved performance in 9% and 35% accordingly has been observed on the square and circle fractal grids in comparison with the mesh-type grid [4]. Therefore, smoothed T-junctions noticeably contribute to a decrease in the viscous dissipation and pumping power.

Similar designs have been installed on a cylindrical surface for efficient cooling of rotating high-speed motorized spindles [70]. The fractal design demonstrated lower pressure drop, more uniform temperature fields, and larger coefficient of performance in comparison to a traditional design with a single channel coiling the same cylindrical shell in a spiral way.

The fractal systems can be optimized at surface, volume, and length constraints [71]. The bifurcation angle was estimated as an important factor during the design optimization of tree-shaped microchannel cooling networks. The fractal systems with acute branching angles produce lower pressure drop along the whole system than that of corresponding parallel straight channels [43,72]. It was shown, at the same boundary conditions, a lower temperature and pressure variations can be obtained at lower bifurcation angles. The pressure monotonously increased along the whole tree when the branching angle increased from 30° to 180°. In [73] for similar Y-junction fractal heat conduction network the best integrated performance was found at *θ* = 60°. The temperature distribution was more uniform for higher numbers of generations. The similar systems with 90° bifurcation angle resulted in a higher pressure drop than that with acute angle bifurcations [48]. A fractal distributor with n=7 generations and branching angle 90° has been tested experimentally and the pressure drop reduction corresponded to 3D CFD computations [74].

In the fractal networks without loops, the increased number of generations decreases the pressure drop and the temperature gradient along the system, while they remain vulnerable for blockage by water drops. Similar fractal geometry with side loops along the perimeter performed significantly better during blockage by using side paths (i.e., loops) for the gas flow and water removal [52].

Disk-shaped heat sinks incorporating tree-shaped fractal microchannels were proposed and then developed [39,75]. They were optimized using an analytical approach [76] for the micro channel heat sinks [55]. A bio-mimetic design proposed for the current collector of a PEMFC showed high current density generation and low pressure drop, but the area covered by the flow channels appeared to limit the FC performance [66]. 3D pyramid configurations of the flow-field plates based on fractal [44] and lung-inspired [33] geometries enhanced the performance of FCs, but they also increased its weight and cost by increasing volume and mass. Besides, this type of FFP was found vulnerable for water flooding [34]. Therefore, a development of a fractal FFP carved in a single graphite plate seems preferable for both lightweight and cheap FC design. 

### 2.2. Flow in Fractal-Like Fluid Delivery Systems

High flow uniformity is essential for FCs and fractal transport systems are interesting for precisely this reason. They can provide uniform delivery of reactants from a point source to a volume. Fractal-like transport systems were first proposed for heat and mass exchangers by A. Bejan [77]. Already at that point, it was realized that geometric optimizations of channel lengths and shapes were necessary [78,79]. The flow in each tube was considered to be in steady state. Then the volume flow rate is

(7) $$Q_{j}=\frac{\Delta p_{j}}{Z_{j}},\;Z_{j}=\frac{k_{j}L_{j}}{d_{j}^{\chi }},$$

where $\Delta p_{j}$ and $Z_{j}$ are the pressure drop along the *j*-th tube and its hydraulic resistivity, and $\chi =4,\;k_{j}=128\mu_{j}/\pi$ for Poiseuille flow in circular tubes, and the viscosity is $\mu_{j}=\mu_{j}(d_{j})$ for non-Newtonian fluids.

In search for a 1D model to fractal channel flows, we replace the real 3D-flow in the channels of different cross-sections by 1D Equation (7). A hydraulic diameter $D_{h}=4A/P$ will be used for each tube instead of $d_{j}$ in Equation (7). Here *A* and *P* are the cross-sectional area and perimeter, respectively. For rectangle channels with width w and depth h, the hydraulic diameter is [80]

(8) $$D_{h}=\frac{2wh}{w+h}.$$

For channels of rectangular, triangular, elliptic and some other cross-sections, analytical solutions (or solutions in expansions) exist. The 2D flow and pressure distributions can be computed for each channel along with corresponding flow rate, viscous dissipation, and entropy production. An analytical expression will reveal the critical properties of the flow field. Until now, however, only simplified 1D approach has been used to estimate the fluid flow along the channels. Then each tube in the fractal pipeline is considered to be infinite and the influence of branching angles, additional pressure loss in the flow developing regions and T- or Y-junctions are neglected [40]. More detailed 3D CFD computations allow quantitative estimations of the flow and temperature uniformity, viscous and thermal dissipation on a confined number of geometries only [81]. 

Due to asymmetry of streamlines at the bifurcations, and relatively short channel lengths, the flow distribution between the outlets of the smallest channels in the closed-channel type fractal flow-fields could be non-uniform: The branching angle (2) that demands minimum power for fluid delivery and distribution may produce a higher non-uniformity of the reactant. 

For a square or rectangular FC, fractal models with α= 90°, for j = 2, 4, 6 generations (T-junctions) and fractal scaling for the width of the channels at a constant depth, are the most likely to give uniform flow distribution and low energy dissipation [36]. For an FFP, as well as for lab-on-a-chip and many other microfluidic flow fields, constant channel depths are to be preferred from a fabrication point of view (e.g., surface micromachining, wet or dry etching, photo-lithography, etc.). Also, it avoids additional pressure loss at the horizontal steps of the junctions in the case of variable depth. In experimental studies [67,68] the FFP was a square plate with side *L* = 32 mm, and channel lengths determined by

(9) $$L_{j}=\{\begin{array}{l} L\cdot 2^{-(j+3)/2},\;j=1,3,5\\ L\cdot 2^{-(j+2)/2},\;j=2,4,6 \end{array},$$

The corresponding channel widths, $w_{j}$, were determined by minimizing the entropy production for constant channel volume. The weighting coefficients α,β were introduced in the optimization function in the form

(10) $$\Phi_{j}=\alpha f_{j}\Delta p_{j}+\beta V_{j},$$

This result is the same as one obtained from Murray’s law (1) derived from (4), and β/α corresponds to Lagrange multiplier.

The pressure drop $\Delta p_{j}$ was computed from Poiseuille’s law using the hydraulic channel diameters (8) at a constant channel depth h. The flow non-uniformity was estimated by the following parameters

(11) $$q=\sqrt{\frac{1}{N-1}\sum_{n=1}^{N}{\left(\frac{Q_{j}}{Q_{avr}}-1\right)}^{2}},\;\theta =\frac{Q_{\max}}{Q_{\min}},$$

where $Q_{j}$ are flow rates in the n=1,…,N terminal channels (i.e., outlets), $Q_{\max}$, $Q_{\min}$, and $Q_{avr}$ are maximum, minimum, and average values of the flow rate, respectively.

The flow uniformity indexes (11) have been computed for a fractal-like design with 16 and 64 outlets, 4 and 6 generation orders, accordingly. The design with 16 outlets had flow uniformity indexes q = 0.001 ÷ 0.055 and θ = 1.003 ÷ 1.014 for Re = 150 ÷ 650. For the same structure, 64 outlets gave a more uniform flow distribution with q = 0.0002 ÷ 0.0013, θ = 1.002 ÷ 1.007 for the same range of Reynold’s numbers. A fractal system with 256 outlets exhibited a very high uniformity of the flow and current distributions, but much higher-pressure drops were needed to drive the same flow, due to a higher resistivity of the more complex FFP with smaller terminal channels [68]. At higher Reynolds numbers, the non-uniformity increased; when Re increased from 1020 to 2247, *q* increased from 0.050 to 0.069 and θ increased from 1.170 to 1.252 [67]. Similar results have been shown by 3D CFD computations for square PEM [36].

## 3. System and Case Studies

The system under special consideration here is a square membrane of dimensions l×l = 5 cm × 5 cm, and the corresponding FFP is carved in-plane into a graphite plate (Figure 2). The fractal pipeline has open channels with $\alpha =180^{\circ }$ and N=4−7 generations. This plate is covered by another plate with the last generation of branches milled into it, located between the first graphite plate and MEA (Figure 1). The dimensions of the openings should provide the Peclet number Pe~1 for better performance [33]. The lengths of the channels are determined by the square design as

(12) $${\left\{L_{j}\right\}}_{j=0}^{N}=\left\{l\cdot 2^{-1},l\cdot 2^{-2},l\cdot 2^{-2},l\cdot 2^{-3},l\cdot 2^{-3},l\cdot 2^{-4},\ldots \right\},$$

The depth of the channels, *h*, is taken to be constant, assuming that this has manufacturing benefits [79]. The widths will be defined as

(13) $$w_{j}=w_{0}\lambda_{j},$$

where $\lambda_{j}$ is the width scaling factor for the *j*-th channel.

The tree designed in the way to provide uniform reactant gas delivery over the square PEM is not self-similar when the lengths (12) are considered. The case $\lambda_{j}=\lambda^{j}$ corresponds to the fractal scaling, and $\lambda =2^{-1/3}$ is according to Murray’s law (1). The number of generations in the tree depends on the scaling factor λ. The size of the terminal channels of the last generation should have a Peclet number near Pe~1 for better performance [33].

The problem of 1D laminar flow in a rectangular duct has an analytical solution [80]

(14) $$v_{x}(y,z)=\frac{4\Delta ph^{2}}{\pi^{3}\mu L}\sum_{n=0}^{\infty }\frac{{\left(-1\right)}^{n}}{{\left(2n+1\right)}^{3}}\left[1-\frac{\cosh\left(\frac{2n+1}{2}\frac{\pi y}{h}\right)}{\cosh\left(\frac{2n+1}{2}\frac{\pi w}{h}\right)}\right]\cos\left(\frac{2n+1}{h}\pi z\right),$$

with volumetric flow rate

(15) $$f(\chi )=\frac{1}{\chi }-\frac{192}{\pi^{5}}\sum_{n=0}^{\infty }\frac{1}{{\left(2n+1\right)}^{5}}\tanh\left(\frac{2n+1}{2}\frac{\pi }{\chi }\right),$$

where χ=h/w is the aspect ratio of the channel. Then, for the constant channel depth h and the given lengths (12), the resistivity of the rectangle channels is determined by their aspect ratios $\chi_{j}=h/w_{j}$ only

(16) $$Z_{j}(\chi_{j})=\frac{12\mu L_{j}}{h^{4}f(\chi_{j})},$$

It was found [36] that the solution (14), (15) approximates 3D CFD data quite well. We consider therefore (14), (15) as a reasonable approximation for detailed computations of the flow parameters and entropy production in different fractal scaling geometries. Additionally, some simplified 1D models for circular tubes are used for direct analytical solution of the optimization problem. 

The following flows were computed from (7) for all channels in the tree-like system:

(1) Flow in rectangular channels computed from (14)–(16);
(2) Flow in cylindrical tubes with equivalent hydraulic diameters $D_{hj}$ determined by (8);
(3) Flow in cylindrical tubes with the same cross-sectional areas $\Sigma_{j}=hw_{j}$ as of the rectangular channels, i.e., with diameters
  (17) $$D_{\Sigma j}=\sqrt{\frac{4hw_{j}}{\pi }},$$
(4) Flow in cylindrical tubes with the same hydraulic resistivity $Z_{j}$ as the rectangular channels, i.e., with diameters
  (18) $$D_{Zj}=\sqrt[{4}]{\frac{8\mu L_{j}}{\pi Z_{j}}},$$

Scaling coefficients $\lambda_{j}$ were computed from the 1D-based expressions for the minimum total entropy production (TEP) and from the state with constant pressure drop along each channel (superscripts S and P accordingly). The minimum TEP approach is common for thermodynamic optimization of engineered devices because it characterizes a system with minimum destruction of available work [82]. The constant pressure drop in the flow system is a good approximation to the state of minimum entropy production [12]. Both computations were therefore done for a tree of a constant TV. This gives two sets of the diameters; $\left\{D_{hj}^{S},D_{\Sigma j}^{S},D_{Zj}^{S}\right\}$ for minimum TEP and $\left\{D_{hj}^{P},D_{\Sigma j}^{P},D_{Zj}^{P}\right\}$ for constant pressure gradient, accordingly.

As a reference case we used the fractal tree scaled according to Murray’s law (6), using the initial rectangle shape (computed on (14)–(16)) and on equivalent cylindrical tubes with $D_{hj}$ (computed on (7)).

This organization of our study allows us to find and use an 1D analytical solution for the different optimization problems, to find the optimal scaling of a cylindrical tube network (with $D_{hj}^{},D_{\Sigma j}^{}$, or $D_{Zj}^{}$), see Section 4. Through this, we will be able to find the best geometry for the FFP in a simple manner.

## 4. The Optimization Problem

### 4.1. The State of Minimum Entropy Production

As it was documented in the literature, see Section 2, optimal designs in nature and engineering have mostly followed from the state minimum entropy production and, thus, maximal efficiency conditions. Therefore, we have also formulated the optimization problem (4) for minimum entropy production at a fixed total channel volume (or its equivalent formulation (5)). As it was shown in [36], the Murray scaling without restrictions on the volume does not produce the overall minimum in the entropy production, but exhibited the most linear pressure fall along the channels of the fractal system. The fixed volume is a reasonable constraint for the flow-field because in the case of a constant depth it corresponds to fixed contact area of the FFP with a gas distribution layer (GDL) (Figure 1). It follows that it is also a condition of fixed electric resistivity of the plate that serves as a charge collector. Therefore, the optimal design determined by (4) will give an optimal solution for FFPs, where also the Ohmic losses are accounted for. Fractal systems with higher scaling factors λ than those in Murray’s scaling are bound to give a slower flow and little viscous dissipation [36]. On the other hand, it makes the contact area of the FFP smaller and, therefore, higher electric resistivity, and ohmic losses. A trade-off situation exists. Therefore, the best design could be chosen based on corresponding Pareto-frontiers.

The TEP in FCs has also significant thermal, chemical and electric sources [83]. This study is limited to the entropy production by viscous dissipation because the FFP is in contact with GDL via the open-end outlets of the FFP only. This is a case which provides a direct analytical solution to the optimization problem. A combined study of all effects may follow. The entropy production in the flow at the isothermal conditions *T* = const is

(19) $${\dot{S}}_{irr}=\sum_{j=0}^{n}\frac{2^{j}\Delta p_{j}Q_{j}}{T}=\frac{Q_{0}^{2}}{T}\sum_{j=0}^{n}Z_{j}2^{-j},$$

When the Poiseuille resistivity is computed on the equivalent hydraulic diameter (8) of the rectangle channel, (19) will have the form

(20) $${\dot{S}}_{irr}=\frac{8\mu Q_{0}^{2}}{\pi h^{4}T}\sum_{j=0}^{n}2^{-j}L_{j}{\left(1+\frac{\chi_{0}}{\lambda_{j}}\right)}^{4}.$$

The necessary condition for convergence of the series in (20)

$$\frac{L_{j+1}}{L_{j}}{\left(\frac{\lambda_{j}}{\lambda_{j+1}}\frac{\chi_{0}+\lambda_{j+1}}{\chi_{0}+\lambda_{j}}\right)}^{4}<1$$

is satisfied when $\lambda_{j+1}/\lambda_{j}<1$ that corresponds to the decreasing width of the tree with increasing the generation number.

Substitution of (20) into (4) gives the following optimization function

(21) $$ℑ(\lambda_{j})=K\sum_{j=0}^{n}2^{-j}L_{j}{\left(1+\frac{h}{w_{j}}\right)}^{4}+\Lambda \pi h^{2}\sum_{j=0}^{n}2^{j}L_{j}{\left(1+\frac{h}{w_{j}}\right)}^{-2}\to \min,$$

where $K=\frac{8\mu Q_{0}^{2}}{\pi h^{4}T}$, and Λ is a Lagrange multiplier.

Murray’s law (1) was initially derived for cylindrical tubes, but the same approach can be applied to the tubes of any shapes.

The necessary conditions for minimum of Equation (21) is

$$\frac{\partial ℑ(w_{j})}{\partial w_{j}}=0,\;j=1,\ldots ,n,$$

This gives the recursive relationship

(22) $$w_{j}=\frac{h}{2^{1/3}(1+h/w_{j-1})-1}\;\;\;.$$

By substituting Equation (22) into the sufficient condition on a positive second order term, we obtain

$$\left|\frac{\partial^{2}ℑ(w_{j})}{\partial w_{j}{}^{2}}\right|>>0,$$

which confirms that the TEP in the fractal system due to viscous dissipation has minimum when the width scaling (22) is used in the design.

The ratio of the cross-sectional areas $\Sigma_{Dh}$ and $\Sigma_{▭}$ of the circular tube with equivalent hydraulic diameter (8) and of the original rectangular tube accordingly is always

(23) $$\frac{\Sigma_{Dh}}{\Sigma_{▭}}=\frac{\pi w_{j}h}{{\left(w_{j}+h\right)}^{2}}<1.$$

Equation (23) shows that hydraulic diameters computed from circular tubes give a smaller cross-sectional area and, therefore, higher resistivity and entropy production. The TEP in a system of cylindrical tubes with diameters $D_{\Sigma j}$ (equal cross-sectional areas of the rectangle duct and its circular model) is

(24) $${\dot{S}}_{irr}=\frac{8\pi \mu Q_{0}^{2}}{Th^{2}}\sum_{j=0}^{n}\frac{L_{j}}{2^{j}w_{j}^{2}}.$$

Substitution of (24) into (4) gives the optimization problem which solution

(25) $$w_{j}=2^{-2/3}w_{j-1}.$$

corresponds to Murray’s law scaling of the cross-sectional area of the cylindrical tubes (6).

The equivalent hydraulic resistivity diameter $D_{Zj}$ model produces the recursive relationship

(26) $$f(h/w_{j})=f(h/w_{j-1})/2^{4/3}.$$

Note that the $D_{Zj}$ -based diameters give in (20) the same hydraulic resistivity of the circular model as it is determined by (16) in the rectangular duct. Therefore, the computation results on (26) really correspond to the analytical formulae (14)–(16) for the rectangular duct with its real dimensions. Therefore, the minimum entropy production at a constant volume approach suggests non-uniform scaling in the form (13). The scaling coefficients depend on the 1D model chosen for analytical calculations of the channels’ widths $w_{j}$ on

Case 1): The equivalent hydraulic diameters (8)—scaling by (22);
Case 2): The equivalent cross-sectional area diameters (17)—scaling by (25);
Case 3): The equivalent hydraulic resistivity diameters (18)—scaling by (26).

For each set of scaling coefficients (22), (25), (26), numerical computations of flow parameters and entropy production on the equivalent circular tube with diameters determined by (8), (17), (18) and on the rectangular flow (13)–(16) determined by (22), (25), (26) will be carried out, for determination of the most energy efficient design. Though the same optimization problem (4) has been used for the scaling coefficients, the TEP and TV produced by the scaling (22), (25), (26) would be different due to the predetermined lower TV (see (23)), higher hydraulic resistivity, electric resistivity and TEP of the $D_{h}$ -based models, and lower resistivity of cylindrical tubes compared to the rectangle ones with the same cross-sectional area. Therefore, the final decision on the best fractal FFP could be made from the results of numerical computations of TEP and TV for each design that will be reported in Section 5.

### 4.2. An Approximation to the State of Minimum Entropy Production: Constant Pressure Gradient

The fractal structure of human lung was found compatible with Murray’s scaling law with the pressure gradient as constant along the flow field [12]. The lowest pressure drop along the flow-field is regarded as one of the key factors of the FFP optimization [36]. Using this result, we may now compute the scaling coefficients for the fractal structure that provides constant pressure gradient along each single pass through it. We assume the flow in each channel is governed by the Poiseuille low (7) with resistivity $Z_{j}$ computed on the circular (7) and rectangular (16) tubes. Then the total pressure drop along the path $\Delta P=p_{0}-p_{n}$, where $p_{0}$ is the inlet pressure, and $p_{n}$ is the pressure at the open end of the terminal tubes of the last generation can be computed as

(27) $$\Delta P=\sum_{j=0}^{n}Q_{j}Z_{j}=Q_{0}\sum_{j=0}^{n}Z_{j}2^{-j}.$$

With $P_{n}=0$, $\Delta P=P_{0}$, the fractal system has the lowest inlet pressure for a given flow $Q_{0}=const$. It follows from (27), that a tree can be designed to obtain a constant pressure gradient value along the channels $\left|\nabla P_{j}\right|=Q_{j}Z_{j}/L_{j}=Q_{0}Z_{j}2^{-j}/L_{j}=const$. This approach corresponds to uniform distribution of the driving force that, according to the theorem of minimal dissipation [84], is a provides a good approach to minimal entropy production design. When the resistivity $Z_{j}$ is computed based on the hydraulic diameter $D_{hj}$, the conditions

(28) $$\frac{\left|\nabla P_{1}\right|}{\left|\nabla P_{0}\right|}=\frac{\left|\nabla P_{2}\right|}{\left|\nabla P_{1}\right|}=\ldots ,$$

give a recursive formula for widths of the channels of different generation number *j*

(29) $$w_{j}=\frac{h}{2^{1/4}(1+h/w_{j-1})-1}.$$

When the resistivity $Z_{j}$ is computed based on the equivalent cross-sectional area (17), the constant pressure gradient condition gives the relationship

(30) $$w_{j}=2^{-1/2}w_{j-1}.$$

When the equivalent hydraulic resistivity diameters (18) are used, the constant pressure gradient approach gives the transcendental equation

(31) $$f(h/w_{j})=f(h/w_{j-1})/2.$$

for computations of the width of the rectangle channels. Now we have three more sets of the non-constant scaling coefficients $\lambda_{j}=w_{j}/w_{0}$ based on the constant pressure gradient approach

Case 4): the equivalent hydraulic diameters (8)—scaling by (29);
Case 5): the equivalent cross-sectional area diameters (17)—scaling by (30);
Case 6): the equivalent hydraulic resistivity diameters (18)—scaling by (31).

Again, in the Case 6), like in the case 3) the computation results will correspond to the flow parameters and entropy production in the rectangular, not circular ducts.

The relationships (22), (25), (26), (29)–(31) have been computed based on the Poiseuille flow in circular tubes with equivalent $D_{\Sigma j}$, $D_{\Sigma j}$, $D_{\Sigma j}$ accordingly, while the scaling is related in each case to the widths $w_{j}$ of the rectangle channels. Therefore, the TEP and TV will be computed based on both rectangular flows (14)–(16) and cylindrical flows (7), (20) for each of the six sets.

Cases 7) and 8): The reference cases on the constant Murray’s scaling coefficient (6) will also be computed on both circular Case 7) and rectangular Case 8) flow models.

Since the condition (28) has been applied to different geometries (rectangular and circular), the resulting pressure gradient would not be the same in each model ($D_{hj}$, $D_{\Sigma j}$, or $D_{Zj}$ -based) because of lower area, higher resistivity and, thus, a higher pressure drop in the $D_{\Sigma j}$ -based models. Besides, the computed values $\left|\nabla P_{0}\right|$, $\left|\nabla P_{1}\right|$, $\left|\nabla P_{2}\right|$ …could not be constant because of the inaccuracy of the computations on (28) based on the rectangular channel and its approximations by cylindrical tubes with equivalent $D_{hj}$, $D_{\Sigma j}$, or $D_{Zj}$ -based diameters. Therefore, the final decision on the best design must use direct 3D CFD computations and/or experimental validation.

## 5. Method of Calculation

### 5.1. Transport Properties

The density and viscosity of oxygen are ρ=1.09 kg/m^3^ and $\mu =2.1\times 10^{-5}$ Pa∙s [36,79]. The flow rates *Q* $=5.71\times 10^{-7}$ m^3^/s and *Q* = $5.71\times 10^{-6}$ m^3^/s correspond to current densities *j* = 1000 A/m^2^ and *j* = 10,000 A/m^2^. These values are typical in FC use. The temperature, often used in FC experiments, was set to *T* = 353 K. The width values for the 0-generation inlet channel have been taken as $w_{0}=1.5;2;3;4;5$, that were used in 3D CDF computations of the same fractal flow field [36] in order to validate the analytical 1D solution.

### 5.2. State of Minimum Entropy Production

Results from Murray’s law’s scaling (6) serve as a reference for all computations. The 1D analytical solutions in circular tubes were validated by more accurate 2D computations in rectangle channels. The latter fit quite well the 3D CFD calculations for the same fractal geometry [36]. The 1D computations were carried out on Equation (19) with the resistivity $Z_{j}$ computed from the Poiseuille formula for circular tubes with minimum TEP at TV=const based on equivalent $D_{hj}$ (Case 1), equivalent $D_{\Sigma j}$ (Case 2), and equivalent $D_{Zj}$ (Case 3) models using (22), (25), (26) accordingly. The constant pressure gradient $\left|\nabla P_{j}\right|=const$ approach has been computed in the same way (Cases 4–6) using (29)–(31) accordingly. As the best approximation to the 3D CFD results [36], TEP values were computed on (19) with hydraulic resistivity (16) for the rectangular channels with the width-scaling relationships (22), (25), (26), (29)–(31).

The aim is to find the best design from the analytical results, knowing that the model is validated by 3D CFD computations on the fractal geometry.

## 6. Results and Discussion

**Figure 3:**
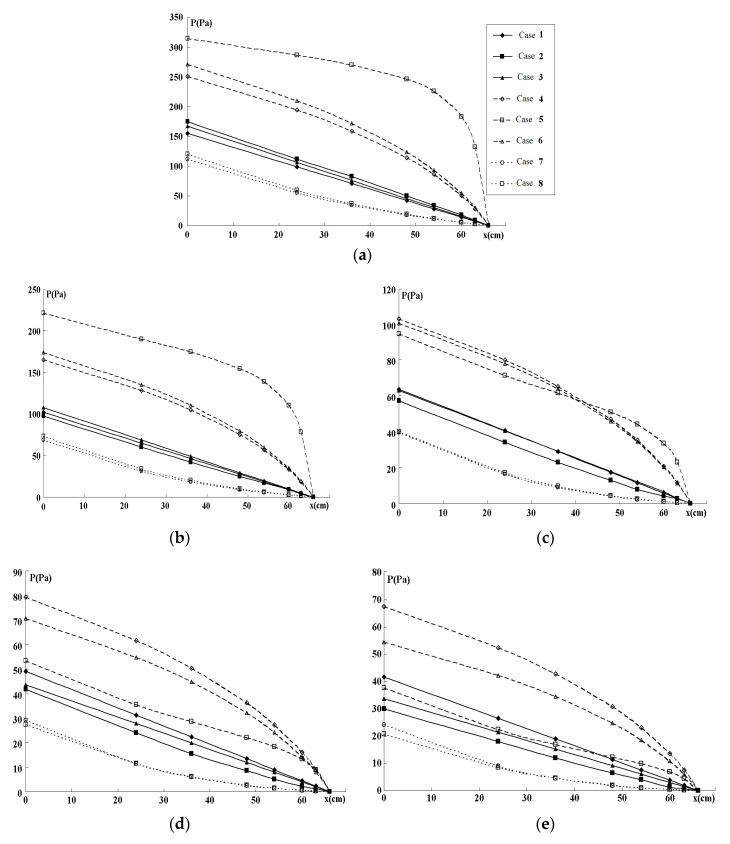
Pressure variation *P(x)* along the fractal-like flow field for five flows with $w_{0}$ = 1.5 mm (**a**), $w_{0}$ = 2 mm (**b**), $w_{0}$ = 3 mm (**c**), $w_{0}$ = 4 mm (**d**), $w_{0}$ = 5 mm (**e**), respectively. The lines correspond to Case (1)–(8) (see explanations in Section 4.1 and Section 4.2).

**Figure 4:**
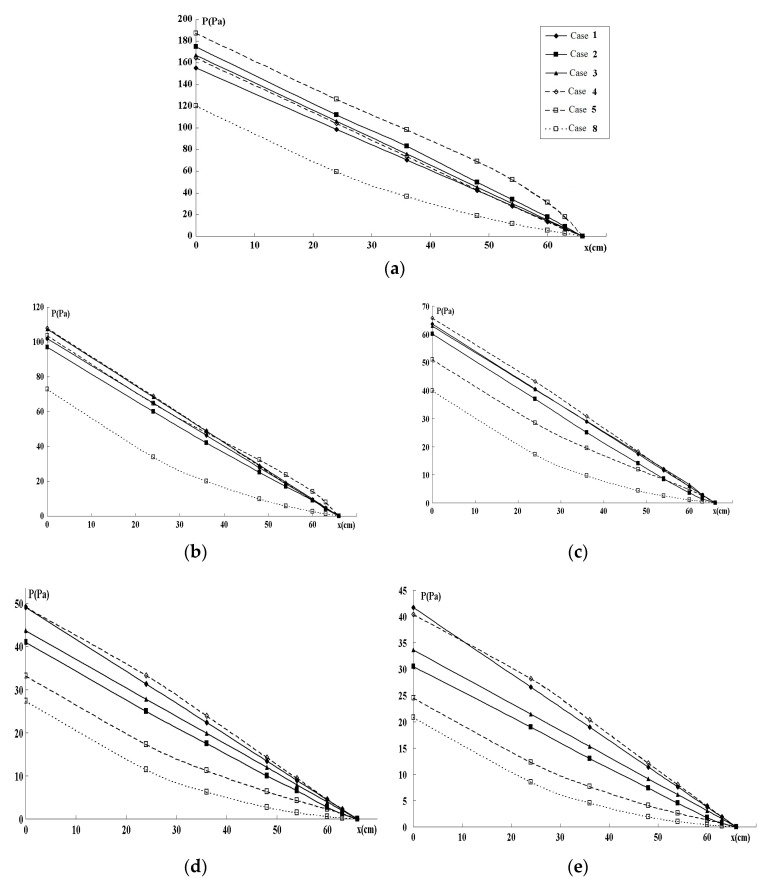
Pressure profiles P(x) along the fractal flow field for five channel widths, $w_{0}$ = 1.5 mm (**a**), $w_{0}$ = 2 mm (**b**), $w_{0}$ = 3 mm (**c**), $w_{0}$ = 4 mm (**d**), and $w_{0}$ = 5 mm (**e**); for the cases 1–8.

**Figure 5:**
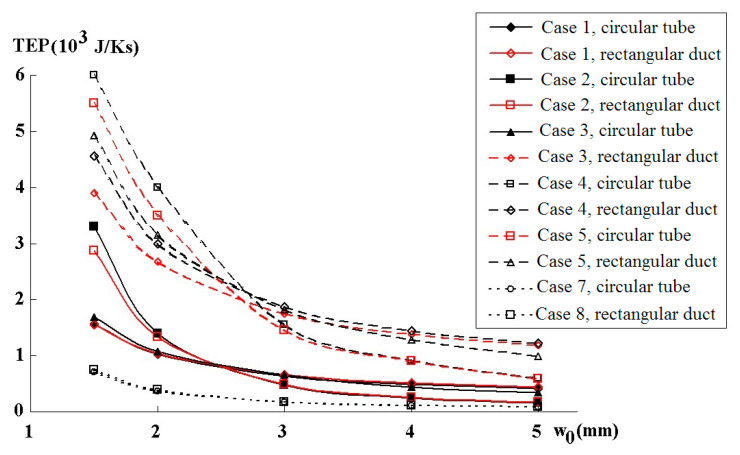
Dependencies TEP($w_{0}$) for the cases 1–5,7,8 computed on the rectangular ducts and their equivalent circular models.

**Figure 6:**
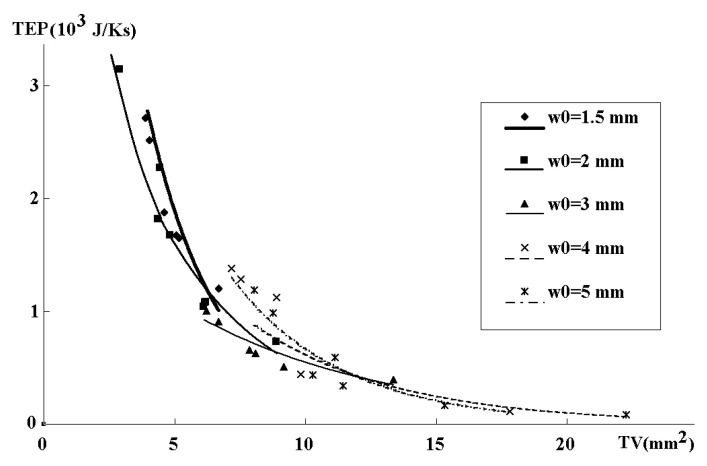
Total entropy production (TEP) as function of total volume (TV) for 1D and 2D systems; the symbols correspond to the cases 1–8 computed on (21), (24), (25), (28)–(30) for the same w_0_.

The numerical results are presented in Figure 3, Figure 4 and Figure 5. Figure 3 and Figure 4 show pressure variations along the generations of six branches for various flows, and channel widths for Cases 1–8. Figure 5 gives the TEP as a function of channel width for Cases 1–8, and Figure 6 gives the TEP as a function of TV for the various scenarios.

The pressure profiles along one pass of the fractal-like system are presented in Figure 3a–e for different values of $w_{0}$ and current density *j* = 10,000 A/m^2^. The variation from the inlet (*j = 0*) to the open end of the last generation (*j* = 6) is shown. The smallest pressure-decrease in the channels was obtained for Murray law’s scaling, and the steepest decrease was when the equivalent area was used to constrain the entropy production.

With the condition |∇P|=const (Cases 4–6) the pressure falls, as expected, linearly (lines 1–3 in Figure 3a–e) and the total pressure drop $\Delta P=P_{0}$ is large at $w_{0}=1.5;2$ and low at $w_{0}=4;5$. This is due to the lower hydraulic resistivity in the bigger channels.

In Cases 1–3, with minimum ${\dot{S}}_{irr}$ the pressure profiles are non-linear and the resulting pressure drop ΔP is always bigger than in the case of |∇P|=const (lines 4–6 in Figure 3a–e). Surprisingly low $P_{0}$ values are demonstrated for Murray-based scaling (lines 7–8 in Figure 3a–e) and the differences between the cylindrical and rectangular tubes are very small in comparison to the differences exhibited by the equivalent $D_{hj}$, $D_{\Sigma j}$, and $D_{Zj}$ computations. In the case of Murray’s scaling, the profile of P(x) is close to linear, in agreement with the 3D computations [36].

In order to find the best 1D approximation to the 2D computations, the pressure profiles along the pass through the fractal system were computed from (15)–(16) for rectangular channels and cylindrical tubes with equivalent $D_{hj}$, $D_{\Sigma j}$, and $D_{Zj}$ at |∇P|=const. The simplified 1D computation on these channel geometries are presented in Figure 4a–e.

In the systems, for all studied $w_{0}$ values, the 1D $D_{hj}$ -based computations on cylindrical tubes give a good correspondence to the 2D flow parameters of the rectangular channels, while the $D_{\Sigma j}$ -based model gives quite poor correspondence. For the inlet channels with the higher aspect ratio $h/w_{0}$ 2D computations give the highest pressure drop $P_{0}$ for the $D_{\Sigma j}$ -based widths, while for the low values $h/w_{0}\leq 1/3$ the pressures in the $D_{\Sigma j}$ -based geometries are lower than in the $D_{hj}$ -based values.

In all Cases (1–8) the lowest pressure drop was detected for the channel with rectangular channel obeying Murray’s law. No significant difference was seen between this and circular channel computations.

The TEP in the fractal system was computed for different $w_{0}$ on the 1D cylindrical tube approximations and the corresponding 2D rectangular channel models with the same scaling values as found with (22), (25), (26) for ${\dot{S}}_{irr}\to \min$ and (29)–(31) for |∇P|=const approaches (Figure 5). In most cases, the rectangular and circular based computations give similar values of TEP when $w_{0}\geq 3$. The correspondence is much better for the |∇P|=const condition than at for ${\dot{S}}_{irr}\to \min$. This means, that the simplified calculations on cylindrical tubes can be used for a first comparison of the performance of different FFP designs.

In the case |∇P|=const, we observe lower TEP values for the equivalent $D_{h}$ -based scaling at $w_{0}<3$ and for the equivalent Σ -based scaling at $w_{0}\geq 3$. Similar regularities appear for the dS/dt→min -approach that produces much higher TEP values. This happens because minimization of the entropy production at a fixed volume also means minimization of the volume at a given entropy production level. The latter leads to a more rapid decrease in the widths of the consecutive channels and, thus, to a rise in their hydraulic resistivity and entropy production. Therefore, the |∇P|=const-based optimization is a preferable tool for lowering the entropy production. Anyway, Murray’s scaling law for the rectangular channel demonstrates much lower TEP values for all $w_{0}$ values. In order to explain this phenomenon, consider the relationships between TEP and total volume TV of the fractal channels presented in Figure 6.

As can be seen from Figure 6, for all values of $w_{0}$, Murray’s scaling produces geometries with lowest TEP and highest total volumes. It is in this situation, that the variation in TEP vs TV can be used to trade geometric variables against each other. The larger TV has the smallest contact area with the gas diffusion layer and, therefore, the higher resistivity and ohmic loss. Another trade-off exists between chemical and electrical power losses. A low-pressure gradient may, however, hamper efficient water removal from the channels. It was recently shown [34], a biomimetic 3D fractal design based on human lung branching rules [33] failed to remove water from the FFP. Therefore, the designs corresponding to the middle locations on the Pareto frontier (Figure 6) with moderate TEP (i.e., not lowest pressure drops) and TV (i.e., with non-significant increase in the FFP electric resistivity) could be more successful that one with minimum entropy production, but increased electric resistivity due to smaller TV.

## 7. Conclusions

Fractal-type flow-fields are beneficial for uniform gas delivery of reactants to catalytic layers, also for the fuel cell. The uniform distribution follows from the position of outlets of the flow field, and from channel geometry: lengths $L_{j}$, widths $w_{j}$, and heights $h_{j}$. To find the optimal scaling of these variables, and define the generations of branches, is central for reduction of viscous dissipation. We show that a simplified analytical expression for 1D model gives results with almost the same accuracy as more detailed 3D computations. The 1D model simplifies the search for optimal geometries, as it can be used directly in an optimization formulation.

The simplified 1D model was obtained, assuming that the rectangular channel in the fractal-like flow field can represent a cylindrical tube with equivalent hydraulic diameter $D_{h}$, or with the same cross-sectional area Σ, or with the same hydraulic resistivity *Z*. Analytical solutions for optimal geometries were found for the state of minimum entropy production for a given channel volume (4) or its equivalent form (5) with minimum TV at a fixed entropy production.

An approximate solution computed from a constant pressure gradient and with the same inlet geometries gives a total dissipation and channel volume. Application of Murray’s law for branch scaling always exhibit the lowest total entropy production (TEP), but at the expense of a total volume (TV) that is higher by a factor two. Therefore, a final decision on non-uniform fractal scaling should use Pareto frontiers of computed TEP vs TV -distributions.

The procedure can be extended to include additional sources of entropy (thermal, chemical, electric). Model validation of the complete flow field with 3D CFD computations and with experiments on the fuel cell is still lacking.

## Figures and Tables

**Table 1:** Summary of experimental and theoretical flow field models in 1D- and 3D CFD.

N	Fuel Cell Pane	FFP Design	Compared to	Verification Method	Heat Effects	Pressure Drop and Pumping Power	Maximum Power Density/Efficiency	Fuel Conversion Rate	FoM*	Wall *T*, Heat Resistivity	*T* Uniformity	Reference
1	Circular	Fractal (n=4, α<π/2),		1D	+	60% ↓	-	-	-	30 °C ↓	-	[39]
2		Fractal (n=4, α=π)		1D+ experiment	+	54–64% ↓	-	-	-	-	2.17–2.78 ↑	[40,41]
3	Rectangle	Fractal (n=5, α<π, smooth) rectangle cross-sections, combined with parallel	Serpentine, parallel	Experiment PEMFC, DMFC	-	similar performance to parallel designs		-	-	-	-	[42]
4	Circular	Fractal (n=4, α<π/2),		3D CFD	+	10% ↓	-	-	-	-	75 ↑	[43]
5	Rectangle	Fractal, rectangle cross-sections		1D	+	8.6–15% ↓	1.7–26% ↑	-	-	-	-	[44]
6	Rectangle	Fractal, rectangle cross-sections		2D	+	↓	↑	-	-	-	-	[45]
7	Rectangle	Fractal (n=5, α=π)		1D	+	↓ for turbulent ↑ for laminar	-	-	-	-	-	[46]
8	Rectangle, Square, Circular	Fractal α=π α=π/2 α<π + parallel				↓	-	-	-	-	-	[47]
9	Rectangle	Fractal (n=2,3, α=π)		3D CFD	+	↓	-	-	-	↓	↑	[37,48,49,50,51,52]
10	Rectangle	Fractal (n=4, α≤π)		1D	+	↑ or ↓	-	-	-	↑ or ↓	-	[53]
11	Square	Fractal (n=6, α=π)		3D CFD	+	↓	-	-	-	↓	↑	[54]
12	Rectangle	Fractal (n=5, α=π)		1D	+	↑ in 5 times	-	-	-	-	↑	[55]
13	Rectangle	Fractal (n=6, α=π)		3D CFD	+	↓	-	-	-	↓	↑	[56,57]
14	Rectangle	Fractal (n=6, α=π)		1D	+	↓	↑	-	-	-	↑	[58]
15	Rectangle	Fractal (n=6, α=π), h=const		3D CFD for DMFC		-	-	10% ↑	-	-	-	[59]
16	Rectangle	Fractal (n=1,2, α=π)		3D CFD	+	↑	-	-	-	↓	↑	[60]
17	Rectangle	Fractal (n=1, α=π/3÷π)		3D CFD	+	↑	30–60% ↑	-	-	↓	↑	[61,62]
18	Rectangle	Fractal (n=4, α=π/2), h=const		3D CFD for DMFC		-	-	↑	↑	-	-	[63]
19	Rectangle	Fractal (n=2, $\alpha =37^{\circ },74^{\circ }$)		3D CFD	-	↓	↓	-	-	-	-	[64]
20	Square	3D 5-layered lung-inspired fractal (n=4, α=π/2 + α=π)	Serpentine	10 cm^2^ FEMFC		↓↓	↑, max in fractal with n=4, better than in serpentine (at 50% and 75% RH**)					[33]
21	Square	3D 5-layered lung-inspired fractal (n=4, α=π/2 + α=π)	Serpentine	Experiment, PEM FC	+	+	↓, high water accumulation; less stable performance than the serpentine	-	-	-	+	[34]
22	Rectangle	Fractal (n=6, α=π)	-	1D, 2D, 3D CFD	-	↓ and close to linear	-	-	-	-	-	[36]

* FoM—the ratio of reactant utilization per unit required pumping power; ** RH—relative humidity; ↓ and ↑ means lower and higher in comparison to the chosen convenient design.
